# A Dynamic Flow Fetal Membrane Organ-on-a-Chip System for Modeling the Effects of Amniotic Fluid Motion

**DOI:** 10.21203/rs.3.rs-4372328/v1

**Published:** 2024-05-15

**Authors:** Sungjin Kim, Po Yi Lam, Lauren Richardson, Ramkumar Menon, Arum Han

**Affiliations:** Texas A&M University; Texas A&M University; The University of Texas Medical Branch at Galveston; The University of Texas Medical Branch at Galveston; Texas A&M University

**Keywords:** Fetal membrane, amniotic fluid, shear stress, microphysiological system, organ-on-chip, preterm birth

## Abstract

Fetal membrane(amniochorion), the innermost lining of the intrauterine cavity, surround the fetus and enclose amniotic fluid. Unlike unidirectional blood flow, amniotic fluid subtly rocks back and forth, and thus, the innermost amnion epithelial cells are continuously exposed to low levels of shear stress from fluid undulation. Here, we tested the impact of fluid motion on amnion epithelial cells (AECs) as a bearer of force impact and their potential vulnerability to cytopathologic changes that can destabilize fetal membrane functions. An amnion membrane (AM) organ-on-chip (OOC) was utilized to culture human fetal amnion membrane cells. The applied flow was modulated to perfuse culture media back and forth for 48 hours flow culture to mimic fluid motion. Static culture condition was used as a negative control, and oxidative stress (OS) condition was used as a positive control for pathophysiological changes. The impacts of fluidic motion were evaluated by measuring cell viability, cellular transition, and inflammation. Additionally, scanning electron microscopy (SEM) imaging was performed to observe microvilli formation. The results show that regardless of the applied flow rate, AECs and AMCs maintained their viability, morphology, innate meta-state, and low production of pro-inflammatory cytokines. E-cadherin expression and microvilli formation in the AECs were upregulated in a flow rate-dependent fashion; however, this did not impact cellular morphology or cellular transition or inflammation. OS treatment induced a mesenchymal morphology, significantly higher vimentin to CK-18 ratio, and pro-inflammatory cytokine production in AECs, whereas AMCs did not respond in any significant manner. Fluid motion and shear stress, if any, did not impact AEC cell function and did not cause inflammation. Thus, when using an amnion membrane OOC model, the inclusion of a flow culture environment is not necessary to mimic any *in utero* physiologic cellular conditions of fetal membrane-derived cells.

## 1. Introduction

Human fetal membrane (amniochorion) lines the intra-amniotic cavity during pregnancy (Menon, Richardson et al. 2019). This membrane is composed of chorion trophoblast cells, amnion mesenchymal cells, and amnion epithelial cells, and plays a significant role in maintaining pregnancy and fetal development (Menon, Richardson et al. 2019, Richardson, Kim et al. 2020). The fetal membranes surround the fetus, enclose amniotic fluid, and protect the fetus during gestation from mechanical and pathological alterations, such as stress, stretch, infection, and inflammation (Manabe, Himeno et al. 1991, Verbruggen, Oyen et al. 2017). The amniotic fluid bathes the innermost amnion epithelial layer and is a shock absorber during fetal movement and growth (1970, Schmidt 1992). Unlike unidirectional blood flow (Martin 1965, Yang 1998, Prefumo, Campbell et al. 2019), amniotic fluid subtly rocks back and forth, and thus, amnion epithelial cells are constantly exposed to shear stress from fluid undulation, but at a very low level (Gilbert and Brace 1993, Modena and Fieni 2004). The ripple effect may impact cells of the amnion membrane; however, amniotic fluid is enriched in growth factors and other nutrients needed for cell survival and growth; hence, any aberrations impacted by the fluid are likely repaired to avoid any disruption in the membrane function (Borgida, Mills et al. 2000). Nevertheless, fluid motion may impact shear stress in conditions like polyhydramnios or oligohydramnios due to abnormal fluid volume impacting membrane function (Sherer 2002, Rabie, Magann et al. 2017). Prior to testing the impact of pathologic fluid volume changes and its motion effect on amnion, a normal baseline understanding of changes that can be induced by fluid motion is needed.

In the human body, physiological shear stress plays an important role in regulating cell differentiation, modulating phenotypes (i.e., epithelial, or mesenchymal), and cellular functionalities (i.e., tight junction expression) based on its frequency, duration, and intensity (Colgan, Ferguson et al. 2007, Anderson and Van Itallie 2009, Cucullo, Hossain et al. 2011, Delon, Guo et al. 2019). During pregnancy, the innermost epithelial cell layer of the fetal membrane is continuously exposed to the amniotic fluid that gently fluctuates by fetal movements (Brace 1997, Brace and Cheung 2011, Brace, Anderson et al. 2014) thus inducing non-uniform levels of shear flow and stress, unlike directional blood flow. However, no *in vitro* model systems have tested the impact of amniotic fluid shear stress on amnion cells.

For the past decades, microphysiological system (MPS), also known as organ-on-chip (OOC), has been developed, validated, and utilized as more physiologically relevant *in vitro* models than conventional 2-dimensional (2D) cell culture platforms, such as well plate or trans-well platforms (Kang, Park et al. 2021, Cao, Zhang et al. 2023). MPS allows better cell-to-cell communications for understanding human physiology and pathophysiology, as well as testing drug efficacy and toxicity, as well as potential harmful effects of environmental toxicants, to name a few examples. Moreover, OOC platforms often integrate microchannels, micropillars, or pneumatic valves to provide biomechanical cues to the cell culture systems such as shear stress, stretch, or contraction that cannot be easily applied in conventional 2D platforms (Cao, Zhang et al. 2023).

In our previously developed amnion membrane (AM) OOC model, using standard culture conditions, we investigated cellular transitions under normal (physiologic) and oxidatively stressed (pathologic) conditions (Richardson, Jeong et al. 2019). The AM-OOC model contained human fetal membrane amnion epithelial cells (hFM_AECs) and amnion mesenchymal cells (hFM_AMCs) separated by type IV collagen filled microchannels modeling the amnion basement membrane, recreating the amnion membrane during human pregnancy. These microchannel arrays allow for the biochemical transportation of signaling molecules from one chamber to another while preventing initial cell mixing between chambers (i.e., although they do allow for active cell migration between chambers due to epithelial-to-mesenchymal transition [EMT]), enabling co-culture of multiple different types of cells. This structure also allows biochemicals such as drugs or environmental toxicants to propagate between cell layers, modeling physiological human pharmacokinetics of stimulants. The use of this platform led to several new findings: 1) amnion membrane cells can migrate (non-reversible) through the microchannels, known EMT and vice versa mesenchymal -to-epithelial transition (MET) and integrate themselves into the emigrated environment; 2) oxidative stress (OS) induces EMT or MET but inhibited migration of cells and pro-inflammatory environment; 3) inhibition of OS by antioxidants and functional inhibitors of stress signaler p38 mitogen-activated protein kinase (MAPK) restore the migration property, suggesting OS and p38 MAPK downstream signaling could regulate cellular migration. This study only focused on visualizing cellular transition and migration; thus, further studies have been proposed such as conducting flow culture to fill the knowledge gap regarding the remodeling of the fetal membrane. Here, we evaluated the effect of amniotic fluid motion on AECs grown within an AM-OOC to glean better insight of *in utero* pathology and pathophysiology induced by oxidative stress.

## 2. Methods

### 2.1 Human fetal membrane cell lines for OOC experiment

We used established immortalized human fetal membrane cell lines as reported in our prior publications (Richardson, Jeong et al. 2019, Richardson, Kim et al. 2020, Richardson, Kim et al. 2020, Radnaa, Richardson et al. 2021, Tantengco, Richardson et al. 2021, Menon and Richardson 2022, Richardson, A et al. 2022, Richardson, Kammala et al. 2023) in our microfluidic *in vitro* model. AECs were cultured in Keratinocyte serum-free medium (KSFM) (17005042, ThermoFisher Scientific, Waltham, MA, USA) supplemented with bovine pituitary extract (30 μg/mL), epidermal growth factor (0.1 ng/mL), CaCl_2_ (0.4 mM), and primocin (0.5 mg/mL). AMCs were cultured in Dulbecco’s modified eagle medium/nutrient mixture F-12 (DMEM/F12) (MT10092CV, ThermoFisher Scientific, Waltham, MA, USA) supplemented with 5% FBS, 10% penicillin/streptomycin, and 10% amphotericin B. All cells were grown at 37 °C and 5% CO_2_ until they reached 80%−90% confluency. Immortalized cells have been validated against primary cells previously (Radnaa, Urrabaz-Garza et al. 2022). Only cells under passage 25 were used for experiments.

### 2.2 Microfluidic OOC model design, fabrication, and experimental setup

**Figure 1:**
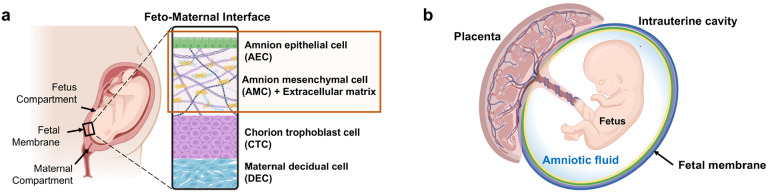
Fetal membrane of the feto-maternal interface and the intraamniotic cavity it surrounds. **a** Illustration of the anatomical structures and cellular components of fetal membrane depicting three fetal cell layers and one maternal cell layer. The highlighted amnion membrane is composed of the amnion epithelial layer and amnion mesenchymal layer. Amnion mesenchymal cells secret type I and III collagen, creating an extracellular matrix layer, as well as organized collagen in the fibroblast layer, tightly packed fibrillar collagen in the spongy layer, loose collagen of the reticular layer. The basement membrane is attached to the underlying chorion trophoblast cells. **b** Amnion membrane lines the human intrauterine cavity that supports fetus development during gestation. During the entire pregnancy, fetus is continuously exposed to the amniotic (shear) fluid surrounded by the amniotic sac and protected from external changes.

**Figure 2:**
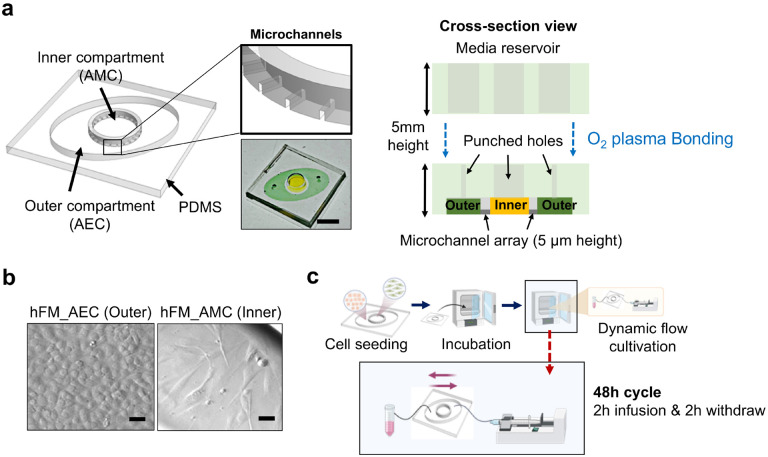
The amnion membrane organ-on-chip (AM-OOC) used for modeling the impact of in vitro amniotic fluid on cells. **a** Illustration of the AM-OOC and a real device image that mimic the amnion membrane. It consists of two cell culture chambers and a media reservoir. The two bottom cell culture chambers are interconnected by an array of microchannel. The top reservoir block is punched to match with inlets and outlet of the bottom cell culture chamber. Scale bar = 1 cm. **b** The two amnion membrane cells (hFM_AECs and hFM_AMCs) cultured in the AM-OOC, observed using bright field microscopy. Scale bar = 25 μm. **c** The experiment flow of creating in vitro amniotic fluid flow that mimics the *in vivo* condition. Syringe pumps and control units are connected to the AM-OOC device, where the device is incubated for cell co-culture whereas the dynamic flow condition is applied.

OOC design and fabrication: To model the amnion membrane (Fig. 1), the AM-OOC device previously developed, tested, and validated to mimic the *in utero* tissue (Richardson, Jeong et al. 2019, Richardson, Emezienna et al. 2022) was modified. Briefly, the device is composed of two cell culture compartments (250 μm height; outer compartment for AECs and inner compartment for AMCs) interconnected by 24 microchannels (5 μm in height, 30 μm in width, 600 μm in length) (Fig. 2a) not only to allow cell-to-cell communication during cultivation and localized biochemical treatment to each compartment, but also to prevent the movement of cells between compartments during the initial cell loading process. The device also contains an on-chip reservoir block where each reservoir compartment is aligned on top of the inlets and outlets of each chamber. The designed platform was fabricated in polydimethylsiloxane (PDMS; Sylgard 184, 1:10 mixture, DowDuPont, Midland, MI, USA) using a two-step photolithography master mold fabrication process, followed by soft lithography process of replica molding (Park, Jang et al. 2009). To improve bonding of the PDMS layer onto the glass substrate, the PDMS layers were treated with oxygen plasma (Harrick Plasma, Ithaca, NY, USA) for 90 s. This process was repeated for the PDMS reservoir bonding onto the PDMS cell culture layer already bonded to a glass substrate.

#### OOC device basement membrane coating

Before cells were loaded into the chip, the microchannels were coated with collagen to mimic the basement membrane of the amnion. Before using the AM-OOC, the devices were washed with 70% ethanol for 15 min for sterilization, washed three times with warm DMEM/F12 media to transit the culture compartment to appropriate culture condition, and then the microchannels were filled with 25% type IV collagen (Corning^®^ Matrigel^®^ Basement Membrane Matrix, Corning, Corning, NY, USA), dissolved in complete DMEM/F12, through an active vacuum suctioning process. The devices were then incubated for 4–12 h at 37 °C and 5% CO_2_ environment.

#### Collection of primary amnion collagen

Methods for obtaining and preparing cell-free collagen from amniotic membrane extracellular matrix (ECM) follow our previously reported procedure (Richardson, Kim et al. 2020, Radnaa, Richardson et al. 2021, Kim, Richardson et al. 2022, Richardson, A et al. 2022).

#### Cell seeding in the AM-OOC

After Matrigel coating of the microchannels, the devices were washed two times with complete DMEM/F12 media before cell seeding. Immortalized AECs and AMCs (Fig. 2b) were trypsinized and loaded into the AM-OOC device (250,000 AECs for outer chamber, 45,000 AMCs cells + 20% primary collagen (Menon, Radnaa et al. 2020) + 25% Matrigel for inner chamber), mimicking the concentration ratios seen *in utero*.

### 2.3 Creating simulated amniotic fluid movement in AM-OOC

To create a simulated amniotic fluid movement environment in the outer compartment of the AM-OOC where AECs are being cultured, a pressure-driven pump controller with 4 independent syringe control units were utilized (HAPC, Harvard Apparatus, Holliston, MA, USA). To mimic the dynamic fluid motion as expected in amniotic cavity (Brace 1997, Modena and Fieni 2004), the pump was programmed to infuse culture media initially from 0 μl/h to a target flow rate, and then maintain for 2 h. Then, the flow gradually decreased to 0 μl/h within 2 min. Once infusion flow stops, flow starts back to opposite direction, maintain the flow for another 2 h, then continue the cycle throughout the 48 h incubation period (Fig. 2c). Flow rate in the outer compartment was converted to shear stress level using the *Navier-Stokes* Eq. (1),

1 $$\tau =\frac{6Q\mu }{{wh}^{2}}$$

where, *τ* is the shear stress, *Q* is the volumetric flow rate, *μ* is the dynamic fluidic viscosity, *w* and *h* indicate the width and height of the channel, respectively.

### 2.4 Creating an oxidative stress (OS) model as pathological positive control

To identify how fluid movement may affect PTB-associated signaling pathways, cigarette smoke extract (CSE) dissolved media was used as positive control to create a pathological condition. A single cigarette (Camel; R. J. Reynolds Tobacco, Winston Salem, NC, USA) was vacuumed into a filtering flask containing 25 mL of hFM_AECs media to obtain 100% CSE. Then, the stock CSE solution was ltered using a 0.22 μm Sterile flip filter (SCP00525, Millipore Sigma, Burlington, MA, USA) and diluted at a 1:25 ratio in the culture media before use. CSE was previously validated as a reproducible OS inducer in our OOC models (Menon, Boldogh et al. 2013, Bredeson, Papaconstantinou et al. 2014, Menon 2014, Menon, Boldogh et al. 2014, Polettini, Richardson et al. 2018, Richardson, Kammala et al. 2023) as well as is a major known risk factor of PTB. After cells have reached to 70–80% of confluency in the AM-OOC, culture media in the reservoir layer was removed and refilled with CSE in the AECs compartment, then incubated for 72 h to model OS condition.

### 2.5 Evaluating the effect of flow culture

The overall effect of flow culture in the AEC and AMC layers was examined by following cellular changes at each culture condition using: (1) lactate dehydrogenase (LDH) cytotoxicity assay, (2) immunocytochemical staining of vimentin, cytokeratin 18 (CK-18), and junction protein E-cadherin, (3) vimentin/CK-18 intensity ratio, (4) cell shape index, (5) cytokine production, and (6) scanning electron microscope (SEM) imaging.

### 2.6 Cytotoxicity assessment

To assess the cytotoxic effect of flow culture on the amnion epithelial cells cultured in the AM-OOC, a LDH cytotoxicity detection kit (ab197004, Abcam, Cambridge, UK) was used. Cell culture media from the cell culture chamber and reservoir in the AM-OOC was collected after the 48 h flow culture experiment. Approximately 5 μL of supernatant were used to perform the cytotoxicity assay according to the manufacturer’s protocol. Briefly, 2 μL of Developer Mix I/LDH Substrate Mix, 4 μL of Pico Probe III/Pico Probe, and 89 μL of LDH Assay buffer were mixed and then added to 5 μL of the collected supernatant in a 96-well plate. The assay plate was incubated at room temperature in the dark for 10 min. The control culture media was used as a negative control, expecting low level of cytotoxicity. Supernatant from cell culture in the AM-OOC was treated with Lysis buffer II/Cell Lysis Solution and incubated for 30 min to ensure lysis of cell membranes as Lysate control. LDH positive control buffer was mixed with 95 μL of prepared LDH Reaction Mix solution to provide positive cytotoxicity. Fluorescence was measured at 535 nm for excitation and 587 nm for emission.

### 2.7 Immunocytochemistry analysis

Immunocytochemical staining for vimentin (1:300; ab92547, Abcam, Cambridge, UK), CK-18 (1:800; ab668, Abcam, Cambridge, UK), and Epithelial (E)-cadherin (1:300; 24E10, Cell Signaling, Danvers, MA, USA) were used to monitor epithelial to mesenchymal transition (EMT) and tight junction expression. Antibodies were titrated to determine the appropriate dilutions to ensure specific and uniform staining. After 48 h, cells were fixed with 4% paraformaldehyde (PFA), permeabilized with 0.5% Triton-X, and blocked with 3% bovine serum albumin in 1X phosphate buffer silane (PBS), before incubation with primary antibodies overnight. Cells were washed three times in 1X PBS and then incubated with species-specific secondary antibodies (1:1000; Goat Anti-Rabbit IgG H&L Alexa Fluor 488, ab150073; Goat Anti-Mouse IgG H&L Alexa Fluor 594, ab150116; Donkey Anti-Rabbit IgG H&L Alexa Fluor 488, ab150073, Abcam, Cambridge, UK) for 1 h. The AM-OOC devices were washed with 1X PBS and then treated with NucBlue^®^ Fixed Ready Probes Reagent (R37606, Thermo Fisher Scientific, Waltham, MA, USA) to stain the nucleus. The concentrations of primary and secondary antibodies were validated based on our previous AM-OOC-based study (Richardson, Jeong et al. 2019).

### 2.8 Microscopy

After 48 h of static and flow culture in the AM-OOC devices, bright field microscopy was performed (BZ-X800E microscope; 4x, 10x, and 20x magnification, Keyence, Osaka, Osaka, Japan) to determine cell morphology, intermediate filament expression, and tight junction.

#### Fluorescence image analysis

More than three random regions of interest per device (N = 5) and per chamber were used to determine overall vimentin, CK-18, and E-cadherin expression. Laser settings, brightness, contrasts, exposure times, and collection settings were uniform for all images collected. Images were not modified (brightness, contrast, and smoothing) for intensity analysis. Image J software was used to measure staining intensity.

Cell shape index (CSI) analysis: CSI was determined for AECs and AMCs by evaluating one frame from each experiment (total of five images) per culture condition for cell circularity using the Image J software. CSI was calculated using the following formula: CSI = 4π × area/perimeter^2^, which is an established method that was originally reported to determine vascular cell shape (Schutte and Taylor 2012). A circle would have a CSI of 1; a straight line would have a CSI of 0.

### 2.9 Scanning electron microscope (SEM) imaging

Cells were fixed by 4% PFA at room temperature for 15 min after 48 h cultivation in static or flow culture condition. Glass substrates were then detached from the PDMS chamber layer and rinsed with 1X PBS three times. A dehydration step was first performed using graded ethanol concentrations: 20%, 30%, 40%, 60%, 70%, 80%, 90%, 95%, and 100%. Cells were immersed in each concentration once for 5 min and twice for 100% ethanol. Samples were gradually transferred to ethanol/hexamethyldisilane (HMDS) mixture in ratios of 3:1, 1:1, 1:3 with 30 min immersion for each step. Samples were then replenished with pure HMDS three times and dried in a chemical fume hood for at least 1 day. A thin gold layer was deposited on samples using a sputter coater (108 Manual Sputter Coater, Cressington, Watford, UK) and imaged with SEM (MIRA 3 Scanning Electron Microscope, TESCAN, Brno, Czechia).

### 2.10 Multiplex assays for inflammatory cytokine markers analysis using Luminex

To assess the changes in inflammatory mediators, interleukin (IL)-6, IL-8, IL-10, and tumor necrosis (TNF)-alpha (α) were analyzed from the cell supernatants in the AM-OOC after 48 h of static or flow cell cultivation. Supernatants were collected from the reservoir of the device using a pipette. Standard curves were developed with duplicate samples of known quantities of recombinant proteins that were provided by the manufacturer. Sample concentrations were determined by relating the fluorescence values that were obtained to the standard curve by linear regression analysis.

### 2.11 Statistical analysis

All data were analyzed using Prism 8 software (GraphPad Software, La Jolla, CA, USA). Ordinary one-way ANOVA analysis of variance followed by Tukey’s multiple comparison test was used to compare normally distributed data with at least three means. Data are shown as mean ± standard error of mean. Asterisks denote *p* values as follows: *p < .05; **p < .01; ***p < .001; ****p < .0001.

## 3. Result

### 3.1 Modeling amniotic fluid movement within the AM-OOC

Fetus drifts in the amniotic fluid during gestation and support fetal organ development, such as lung, digestive system, and protect from bacterial infection and environmental alterations, such as temperature change, and injury (Shamsnajafabadi and Soheili 2022). It also allows the movement of fetus, which permits the musculoskeletal development (Fitzsimmons and Bajaj 2023). Unlike other flow *in vivo* as summarized in Table 1, amniotic fluid can be influenced by various situations, such as reduction of the amount of amniotic fluid through swallowing (Ross and Nijland 1997) and absorbance (Brace and Cheung 2011) of fetus, or maternal abdomen trauma. We established experimental set up for fluid motion by utilizing programmed pressure-driven pumps and generated two distinctive flow conditions, representing a moderate flow (i.e., 50 μl/h; 5.3 dyne/cm^2^) and a much higher flow condition (i.e., 200 μl/h; 22 dyne/cm^2^). Specifically, after the cells have reached 70–80% confluency in the AM-OOC, a 3 mL syringe (Luer Lock, BD, Franklin Lakes, NJ, USA) containing complete KSFM media was placed on the syringe control unit and then tubing (EW-06420, Tygon, Irvine, CA, USA) was plugged to both the syringe and the inlet of the device to start a flow within the outer compartment for continuous shear force application. The tubing, connected to the outlet of the outer culture compartment, was immersed in a complete KSFM media in 15 mL conical tube to avoid air bubbles coming into the device during the entire flow culture condition.

### 3.2 Low levels of shear stress do not induce AEC cytotoxicity

**Figure 3:**
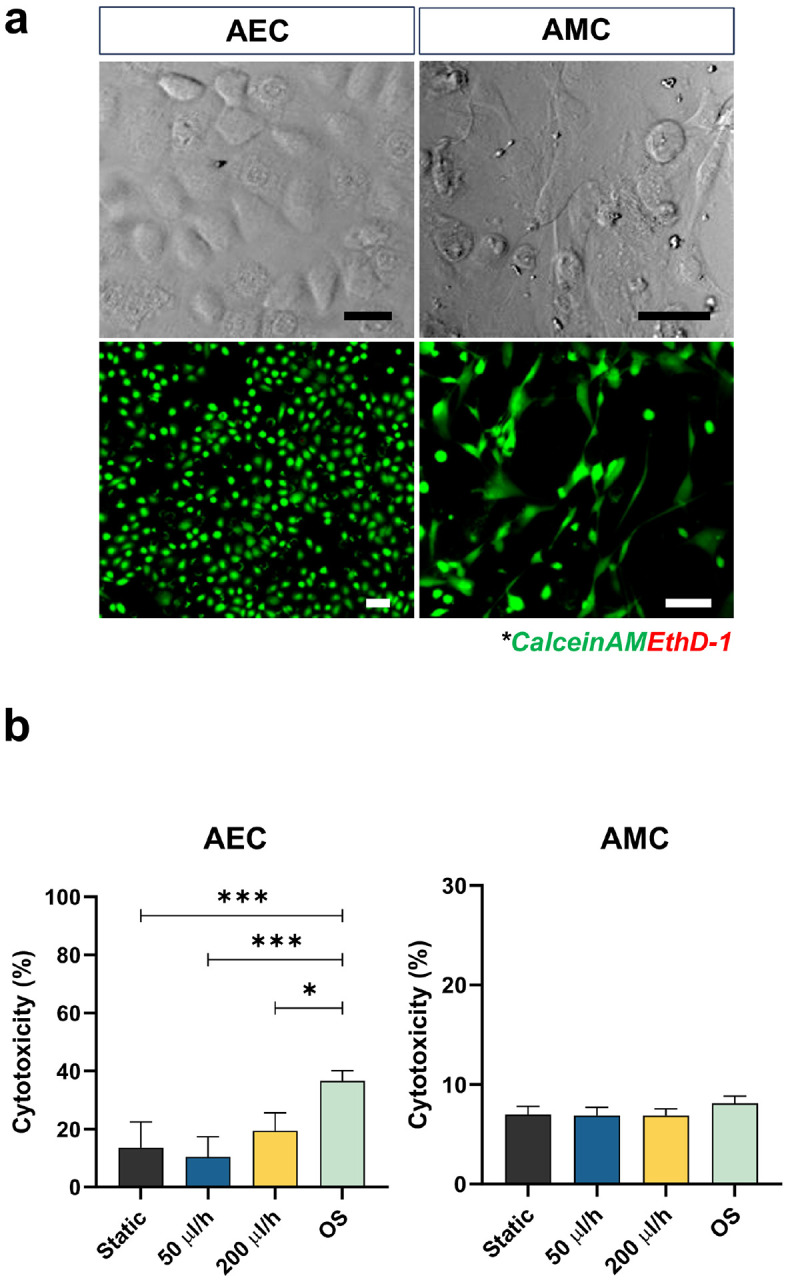
Characterization of amnion membrane cells cultured by different conditions for 48 h in the AM-OOC. **a** Bright field and fluorescent microscopy showing normal condition of amnion cells in AM-OOC with high viability. The scale bar is 10 μm. **b** LDH cytotoxicity assay result showing the percentage of dead AECs and AMCs in the AM-OOC after 48 h culture. Data are shown as mean ± SEM (N=5).

AEC and AMC viability was assessed by calcein acetoxymethyl (AM) (Live; green) and ethidium homodimer (EthD)-1 (Dead; red) staining after 48 h of on-chip cultivation. Both cell types showed good viability, with calcein AM expression and proper cell morphology (i.e., AEC – cuboidal; AMC – fibroblastoid) (Fig. 3a). AECs had low levels of cytotoxicity (< 20%) under both static and fluid flow conditions, while OS induced significant levels of cytotoxicity (AEC - static vs. OS: p = 0.0004; 50 μl/h vs. OS: p = 0.0004; 200 μl/h vs. OS: p = 0.0269), but AMCs showed no significant change (Fig. 3b). These results confirmed that the applied flow and associated shear stress did not induce cell cytotoxicity, whereas the control experiment with OS induced cytotoxicity only in AECs as expected.

### 3.3 Flow does not affect the epithelial nature of AECs but does induce microvilli formation

Highly elastic (due to rich presence of elastin) AECs protect the fetus by forming a watertight barrier between the amniotic fluid and the distal cellular layers (Shamsnajafabadi and Soheili 2022, Truong, Menon et al. 2023). This occurs through its maintains of epithelial characteristics such as intermediate filament organization, tight junction regulation, and microvilli expression (Anderson and Van Itallie 2009, Kobayashi, Kadohira et al. 2010).

**Figure 4:**
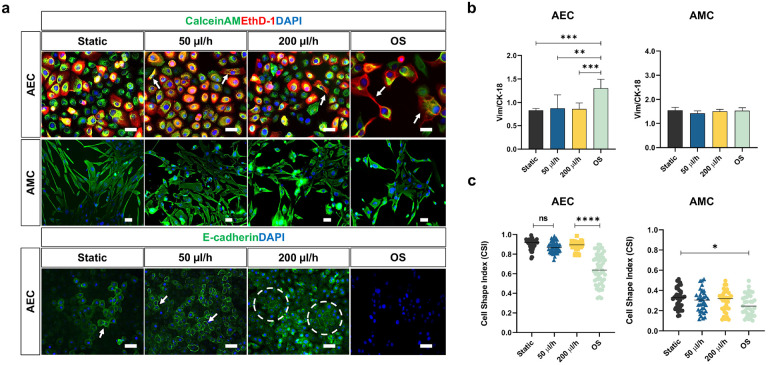
Evaluating amniotic fluid motion on amnion membrane cells in AM-OOC. **a** Fluorescence microscopy showing the expression of in utero amnion membrane cell specific markers after 48 h culture. These measurements included cytoskeletal marker (vimentin; green and cytokeratin-18 [CK-18]; red) of AECs and AMCs on the top and cell to cell junction protein marker (E-cadherin; green) of AECs on the bottom. The scale bar is 10 μm. **b** Intensity analysis of immunocytochemical cell specific markers documented minimal changes in expression by flow over 48 hr with different culture conditions on-chip. Data are shown as mean ± SEM (N=5). **c** Amnion membrane cells morphological changes and cellular transition were determined by cell shape index analysis. AECs under static culture condition averaged cell shape index of 0.9 correlated to its cuboidal nature, while AECs under OS condition average significantly decreased index. AMCs did not show significant morphological changes under dynamic flow culture. Data are shown as mean ± SEM (N=5).

After 48 h of static and flow culture conditions, intermediate filament expressions (vimentin for mesenchymal characteristics; CK-18 for epithelial characteristics) and localization in AECs and AMCs were evaluated by immunocytochemistry. AECs under all conditions co-expressed both vimentin and CK-18, highlighting that they were in “meta state”, an in-between state of cellular transition that is an innate characteristic of AECs in utero (Martin, Richardson et al. 2018) (i.e., vimentin; green, CK-18; red, and DAPI; blue; Fig. 4a). Static and flow cultures did not affect vimentin or CK-18 expression, as seen by non-changing vimentin/CK-18 ratio levels (Fig. 4b). However, OS treatment did induce vimentin re-localization toward the leading edge of cells (white arrow) and a significant increase in vimentin/CK-18 ratio compared to all other conditions (Fig. 4a and 4b) (AEC - static vs. OS: p = 0.0008; 50 μl/h vs. OS: p = 0.0013; 200 μl/h vs. OS: p = 0.0008). None of the culture condition affected intermediate filament expression and localization in AMCs. Morphological changes of AECs and AMCs evaluated by cell shape index (CSI) analysis (Fig. 4c) (AEC - static vs. OS: p = 0.0001; 50 μl/h vs. OS: p = 0.0001; 200 μl/h vs. OS: p = 0.0001, AMC - static vs. OS: p = 0.02142) confirmed the findings of no intermediate filament expression changes, suggesting that fluid flow does not induce cellular transitions (i.e., EMT) in AECs. In contrast, OS treatment for 48 h (positive control) does induce vimentin re-localization, increase in vimentin/CK-18 ratio, and increase in fibroid morphology (high CSI), therefore indicating EMT in AECs. This confirmed our prior reports where we showed that CSE-induced OS in AEC can cause EMT (Richardson, Jeong et al. 2019).

E-cadherin was evaluated by immunocytochemistry to measure barrier function and tight junction expression between AECs within the AM-OOC. AECs under static and flow culture conditions stained positive for E-cadherin (green) but not in OS condition, and flow culture induced some increase in E-cadherin expression with more group of epithelial morphologies (Fig. 4a, E-cadherin: green, DAPI: blue).

**Figure 5:**
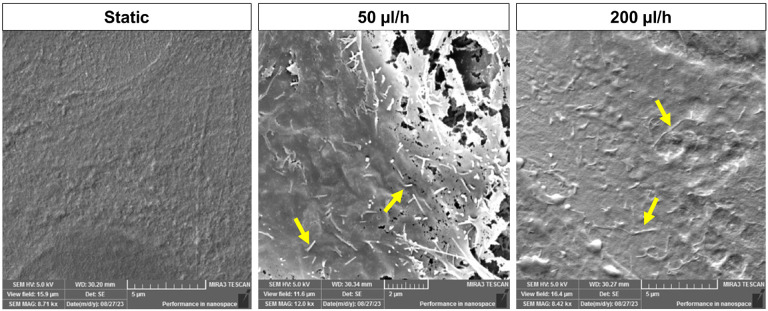
Scanned electron microscopy (SEM) image of hFM_AECs after 48 h. SEM technology was utilized to see the microvilli formation from shear flow. Microvilli formation was observed from flow culture while static culture condition did not show any changes after 48 h in the AM-OOC.

Microvilli expression on-chip was assessed by SEM. After 48 h culture, static culture did not induce microvilli formation, while fluid flow induced microvilli expression on the apical surface of the AECs (Fig. 5), as seen in *in utero* (Basile, Centurione et al. 2023) and seen in typical epithelial cells as well (Zhao, Chien et al. 2001, Miura, Sato et al. 2015).

In summary, these results indicate that fluid flow does not affect AEC’s epithelial properties (i.e., cuboidal morphology [high CSI], meta state [no change in Vim/CK-18 ratio], and tight junction expression [E-cadherin]) but produced microvilli of AECs. Microvilli are expressed in absorptive epithelial cells but not necessarily required feature from cells in 2D culture or in a static state as they supplemented nutrient directly from culture media unlike cells in 3D environment where absorb material from the fluid flowing through, and thus their appearance in a flow environment is an expected phenomenon.

### 3.4 AECs do not change cytokine production due to fluid flow rate

**Figure 6:**
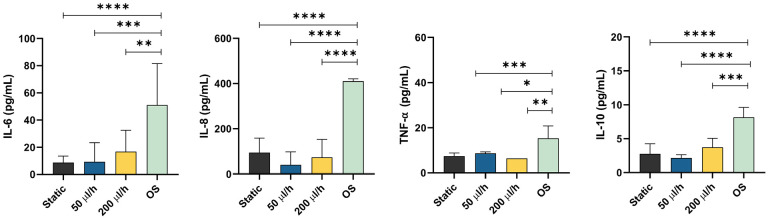
Production of inflammatory mediators by different culture conditions in the AM-OOC. Interleukin [IL]-6 showed flow-dependent inflammation and IL-8 from AM-OOC showed increased inflammatory responses compared to 2D condition, but not significant. TNF-α and IL-10 did not show any changes among all conditions. Regardless of the culture condition (static vs. flow) and flow rate (50 μl/hr vs. 200 μl/hr), amnion epithelial cells do not produce inflammatory mediators for 48 h culture compared to 2D control static culture (p>.05) and OS condition (p=0.00001). Data are shown as mean ± SEM (N=5).

Pro- and anti-inflammatory cytokines (i.e., IL-6, IL-8, TNF-α and IL-10) were measured after 48 h to determine if fluid flow induced a pro-inflammatory status in AECs (Fig. 6). As reported in the previous study (Richardson, Jeong et al. 2019), AECs within the AM-OOC expressed higher cytokine values than 2D culture alone, likely due to the presence of AMCs in proximity (Fig. 6). Neither low (50 μl/h) nor high (200 μl/h) levels of fluid flow significantly increased IL-6, IL-8, TNF-α, and IL-10 production compared to static culture. While OS treatment significantly increased the production of pro- and anti-inflammatory cytokines compared to static and fluid flow conditions (IL-6: static vs. OS: p = 0.0001; 50 μl/h vs. OS: p = 0.0005; 200 μl/h vs. OS: p = 0.0073) (IL-8: static vs. OS: p = 0.0001; 50 μl/h vs. OS: p = 0.0001; 200 μl/h vs. OS: p = 0.0001) (TNF-α: static vs. OS: p = 0.0008; 50 μl/h vs. OS: p = 0.0238; 200 μl/h vs. OS: p = 0.0052). (IL-10: static vs. OS: p = 0.0001; 50 μl/h vs. OS: p = 0.0001; 200 μl/h vs. OS: p = 0.0003). Together, these results confirm that fluid flow does not induce pathologic inflammatory imbalance within AM-OOC cultures.

## 4. Discussion

Human body is consisting of up to 60% water, which circulates inside and outside the cells by constituting intracellular and extracellular fluid. Blood circulates human body and acts on the endothelial surface of vessels by inducing shear stress. This mechanical phenomenon impacts various biological functions and processes. Thus, various types of *in vitro* models have been developed with flow-based cultures to recreate organ- or tissue-specific phenomenon and conditions that mimic the *in vivo* environment. However, amniotic fluid behaves differently and its role and impact in microphysiological system of feto-maternal interfaces have not been previously studied, due in part lack of information on the *in vivo* fluid conditions. Amniotic fluid fluctuates during gestation from 1–1.5 ml in the first week to 1.5 L at term. The maintenance of fetal and fetal organ growth as well as amnion cell nourishing is performed by the nutrient and growth factor enriched in amniotic fluid. The low (oligohydramnios) and high (polyhydramnios) amniotic fluid volume is pathologic and associated with pregnancy-associated adverse pregnancy outcomes such as growth restrictions and preterm birth (Fortunato, Menon et al. 2004, Kacerovsky, Musilova et al. 2014). Under these conditions, the flow, motion and stress are expected to differ, which can contribute to cytopathologic changes to amniotic cells.

Amniotic epithelial cells undergo multitudes of changes during pregnancy, which include senescence, EMT, and autophagy. All these are natural and physiologic processes required for membrane integrity. However, premature senescence, terminal state of EMT (i.e., lack of MET to recycle cells), and accumulation of autophagosome complexes or incomplete autophagy are pathologic process that destabilizes membranes and predispose them to adverse events. Stress, stretch, scratch, and senescence can all cause change in amnion functions inducing inflammation. The impact of fluid and its motion is aiding membrane function; however, under pathologic environment, the impact of fluid motion on amnion cells and fetal responses are unknown due to difficulties in studying such phenomenon *in vivo*.

The study presented here established and *in vitro* experimental model to recreate amniotic fluid motion and determined the effect of amniotic fluid motion on fetal epithelial layer. We utilized a pressure-driven pump providing a back and forth flow operation to mimic the rocking movement, recapitulating amniotic fluid motion for the first time in an organ-on-a-chip model. The use of this model was used to provide better insight into fetal membrane homeostasis during pregnancy. We report that shear stress has minimal impact on amniotic epithelial cells. Specifically, regardless of the flow rate, AECs did not undergo cytotoxic changes, exhibited expected morphology, metastatic status, absence of overabundance of transitioning cells with mesenchymal morphology, and did not produce inflammation. Although not measured, lack of inflammation is also indicative of absence of senescence-associated secretory phenotype (SASP), an inflammatory signature associated with amnion epithelial cell senescence. These data are reassuring that the motion induced by amniotic fluid is not expected to cause any cytopathic changes in AECs.

Using the microfluidic OOC platform enables overcoming the limitations of conventional cell culture platform, such as lack of multicellular communication, inadequate physiologic environment, applicability of various operational components (such as pump and valves) that control the cellular culture environment. Incorporating flow dynamics into OOC platform has helped better understand endothelial and epithelial cell behavior and recreating vasculature flow and relevant fluidic kinetic studies as it mimics more realistic environment. Here, rocking amniotic fluid motion was established through a syringe pump system by programming the infusion and withdraw function (2 h back and forth cycle for 48 h) with different shear stress level. One of the limitations of this study is that the actual level of amniotic fluid motion is unknown, as *in utero* this motion is primarily affected by the movement of fetus. Therefore, it fiuctuates all the time, which is difficult to exactly mimic. In addition, in this study we have used regular AEC culture media instead of that mimicking amniotic fluid. Thus, changes in amniotic fluid constituents under normal and pathologic events and fetal movement under hostile intrauterine environment was not adequately replicated in this model. These factors may impact AECs functions not studied in this work, leaving such studies to be conducted in the future.

In conclusion, we established an easy and simple amniotic fluid motion organ-on-a-chip model by applying moderate to high level of shear flow to the amnion epithelial layer and monitored their cellular responses. Within the AM-OOC device, AECs maintained their phenotypic expressions without cellular transition under all flow culture conditions and did not show inflammatory responses commonly seen in pathologic condition. This indicates that pregnancy maintenance and fetal development is not affected by mechanical stimulations induced by fluid motion in the intrauterine environment under normal circumstances. Furthermore, this study provides evidence that an inclusion of shear flow within amnion layers is not a requirement when studying amniochorion functions using OOC.

## Figures and Tables

**Table 1:** Summary of in vivo Shear Flow in Human Body

Organ/tissue	Fluid shear stress (dyne/cm^2^)	Ref.
Brain arterial vessels	10–70	(Wang, Xu et al. 2020)
Brain capillary network	5–23	(Wang, Xu et al. 2020)
Renal	0.3–1.2	(Ross, Gordon et al. 2021)
	2–4	(Wang, Gust et al. 2022)
Glomerular tubule	30–50	(Wang, Gust et al. 2022)
	5–20	(Ballermann, Dardik et al. 1998)
Podocyte slit diaphragm	80	(Wang, Gust et al. 2022)
Alveolar	0.5–3	(Denise C. Fernandes 2018)
Intestinal tract	0.002–0.08	(Denise C. Fernandes 2018)
Large veins	<1	(Ballermann, Dardik et al. 1998)
Small arterioles	60–80	(Ballermann, Dardik et al. 1998)
Placenta	0.04–0.2	(Lee, Moulvi et al. 2023)

## Data Availability

The data that support the findings of this study are available on request from the corresponding author.
